# Gender differences in sleep quality among Iranian traditional and industrial drug users

**DOI:** 10.1016/j.nbscr.2024.100104

**Published:** 2024-07-02

**Authors:** Mohammad Khorrami, Fatemeh Khorrami, Kosar Haghani, Farshid Fathy Karkaragh, Ayda Khodashenas, Sara Souri

**Affiliations:** a Islamic Azad University, Tehran Science & Research Branch (Isfahan), Isfahan, Iran; b Faculty of Paramedicine, Golestan University of Medical Sciences, Gorgan, Iran; c Department of Social Sciences University of Mazandaran Babolsar, Iran; d Health Psychology, Department of Health Psychology, University of Tehran, Tehran, Iran; e Clinical Psychology, Department of Psychology, Ferdowsi University of Mashhad, Mashhad, Iran; f General Psychology in Payam Nour University, Branch Amol, Amol, Iran

**Keywords:** Sleep quality, Gender differences, Traditional drug users, Industrial drug users, Substance users

## Abstract

• The findings emphasize gender differences in sleep quality among different cultures, races, and ethnicities.
• There are gender differences in sleep quality in Iranian traditional and industrial drug users.
• Industrial drug users have a lower quality of sleep than traditional drug users.
• The sleep quality of drug users (traditional and industrial) is lower than that of healthy people.

There is a significant research gap in understanding how sleep quality intersects with gender differences in drug addiction. This study addresses this gap by examining changes in sleep problems between genders among Iranian traditional and industrial drug users. We hypothesized that drug users would experience sleeping problems and that women would report worse sleep than men. Using a descriptive-analytical cross-sectional design, this study focused on male and female members of Narcotics Anonymous (NA) in Bojnord city in 2012. Through convenience sampling, 115 drug users were selected, and data were collected from a control group of healthy individuals matched on demographic factors. Sleep quality and problems were evaluated using the Pittsburgh Sleep Quality Index (PSQI). Data analysis was performed using descriptive statistics, two-way analysis of variance, and independent two-sample t-tests to test the hypotheses. In terms of gender, women in all three groups showed poorer sleep quality than men, with statistical significance found only in the industrial substance user group. When comparing the studied groups, both women and men in the traditional and industrial drug user groups showed significantly higher mean scores in the seven scales and the total sleep quality score, indicating poorer sleep quality compared to their healthy counterparts. However, there was no significant difference in sleep quality between traditional and industrial drug users. These findings emphasize significant gender differences in sleep quality among different cultures, races, and ethnicities (see Fig. 1).

**Fig. 1:**
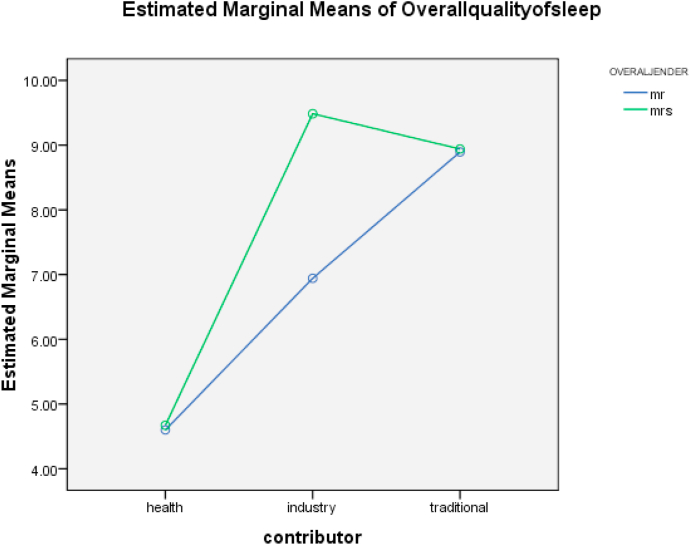
Mean sleep quality scores by group and gender.

## 1 Introduction

Sleep plays a pivotal role in regulating brain function and maintaining physiological homeostasis across various bodily systems (Knoop et al., 2021). It actively contributes to essential processes, including growth, tissue repair, facilitating learning, and consolidating memory (Schimke et al., 2021). Indeed, sleep quality is intimately linked to human health (Hepsomali and Groeger, 2021). Conversely, sleep-related problems are highly prevalent, encompassing deficits in sleep quantity and quality (Huyett et al., 2021). The constellation of issues affecting sleep continuity is collectively termed sleep disorder (Medic et al., 2017). Sleep disorders manifest as insomnia (e.g., sleep apnea and narcolepsy), parasomnia (restless leg syndrome and sleepwalking), and sleep-wake cycle disturbances (Ciren et al., 2021), which can adversely impact concentration, alertness, and memory (Cruz et al., 2021). Non-restorative sleep and hypersomnia are associated with respiratory, cardiovascular, and neurological complications, as well as increased reaction time, potentially leading to road hazards and serious accidents in situations demanding high alertness (Jennum and Kjellberg, 2011; Laaban et al., 2010; Punjabi et al., 2009). Consequently, sleep disorders pose a significant threat to overall health (Schipper et al., 2021), exerting profound effects on individuals' emotional well-being, psychological state, and overall quality of life (Nasri et al., 2021; Benbir et al., 2015).

Sleep disorders affect a substantial portion of the general population (Baranowski and Jabkowski, 2023). A systematic study revealed that approximately 4–26% of individuals suffer from sleep disorders (Ohayon, 2011). The prevalence of sleep disorders, such as obstructive sleep apnea and restless legs syndrome, increases with age (Brewster et al., 2018; Gulia and Kumar, 2018; Chowdhuri et al., 2018), resulting in a relatively high incidence among adults, which is associated with elevated mortality rates (Tang et al., 2017). Notably, 70% of participants in a 2005 National Sleep Institute survey reported that their treating physicians had never inquired about their sleep habits or patterns, nor investigated the issue (Sleep in America Poll - Summary of Findings, 2005).

Sleep disorders are closely intertwined with psychological, medical, and social disorders (Nowowiejska et al., 2021; Vgontzas and Kales, 1999). Sleep problems are associated with most mental disorders, including anxiety, depression, bipolar disorder, attention deficit hyperactivity disorder, alcoholism, and other substance use disorders (Terán-Pérez et al., 2020; Marvaldi et al., 2021; Roehrs et al., 2021). Drug use is a complex phenomenon involving various factors, such as emotional states, interpersonal relationships, environmental influences, and physical symptoms (Kaviyani et al., 2023), and its relationship with sleep disorders is bidirectional (Edwards et al., 2015). Nearly 70% of patients hospitalized for substance abuse detoxification report sleep problems (Roncero et al., 2020). A high prevalence of sleep disturbances has been documented among chronic drug users, including methamphetamine, heroin, and ketamine users (Li et al., 2018; Tang et al., 2015a, Tang et al., 2015b, Tang et al., 2015c; Chen et al., 2017), as well as individuals with behavioral addictions, such as Internet addiction (Alimoradi et al., 2019) and Instagram addiction (D'Souza and Negahban, 2019). Furthermore, the effects of other illicit substances, including methamphetamine, cocaine, ecstasy, and marijuana, have been investigated and confirmed using polysomnography (a test that electronically records specific bodily activities during sleep) to impair sleep quality. For instance, cocaine administration suppresses rapid eye movement (REM) sleep and increases wakefulness (Schierenbeck et al., 2008).

Numerous studies have indicated a correlation between chronic drug use and sleep-disordered breathing (Mubashir et al., 2020; Correa et al., 2015; Wang and Teichtahl, 2007; Farney et al., 2003). The results of a meta-analysis study showed that the increased risk of respiratory depression associated with opioid use is related to the presence of obstructive sleep apnea in individuals. Opioids are also associated with abnormal breathing patterns, such as Biot's respiration or ataxic breathing, hypoxemia, and hypercapnia. The exact mechanism by which opioids cause sleep-disordered breathing is not fully understood (Correa et al., 2015). During another study, a high prevalence of central sleep apnea was observed in patients who used narcotic drugs for chronic pain and addiction (Mubashir et al., 2020). The pathophysiology of central sleep apnea caused by opioids is attributed to disturbances in the production of respiratory rhythm and respiratory chemical reflexes. Opioids exert paradoxical effects on different brain regions, leading to irregular respiratory rhythms (Wang et al., 2021).

Therefore, the authors are addressing three hypotheses in their present study.

(1) Sleep is more disturbed in drug users than non-drug using subjects;
(2) Women report more sleep disturbances than men;
(3) There will be a difference between sleep disturbances in traditional opioids and industrial users.

## 2 Gender differences in sleep problems among healthy individuals and drug users

Sleep disparities between men and women manifest at an early age, with women reporting poorer sleep quality and being at greater risk for insomnia disorder compared to men (Petersen et al., 2023). Sleep patterns can be influenced by gender-related factors, including hormonal fluctuations, physical and mental health conditions, life transitions, the aging process, and other contributing biological and environmental factors. Females, in particular, are more susceptible to chronic insomnia, especially during phases of reproductive hormonal changes. The menstrual cycle is associated with alterations in circadian rhythms and sleep architecture, with women often reporting poorer subjective sleep quality and increased sleep disturbances in the late luteal phase preceding menstruation. Pregnancy introduces common sleep disorders, escalating in prevalence and severity as gestation progresses. Postpartum, the hormonal fluctuations and erratic sleep-wake patterns of newborns can exacerbate maternal sleep disturbances. Menopausal women commonly experience insomnia symptoms, with decreasing testosterone levels in aging men also associated with poorer sleep during this phase. The aging process affects the sleep of men and women differentially (Meers et al., 2019).

In subjective self-report studies, women consistently report more sleep complaints, poorer sleep quality, and are at greater risk for insomnia disorder than men across a wide age range. Men and women also subjectively interpret and perceive the quality of sleep differently. In contrast, objective measures of sleep, such as polysomnographic (PSG) studies, indicate that women objectively exhibit better sleep (e.g., longer total sleep time, fewer awakenings, higher slow-wave sleep), and the age-related decline in sleep quality has a smaller effect on PSG measures in women compared to men. This suggests that objective and subjective assessments may capture different aspects of sleep experiences (Baker et al., 2020; Bixler et al., 2009).

Moreover, sleep disturbances among drug users exhibit unique gender-specific patterns, differing within each gender and compared to non-substance using individuals. The results of a study on 2178 illegal drug users showed that overall, men and women had prevalence rates of sleep problems at 67.4% and 75.2%, respectively. Among methamphetamine users, 52.4% were men, and 75.6% were women. For heroin or other opioid drug users, 80.8% were men, and 75.1% were women. The study assessed sleep quality using the Pittsburgh Sleep Quality Index (PSQI), with average total scores indicating poorer sleep quality for all drug users, particularly men using heroin or other opioids, compared to healthy control subjects. Interestingly, women using methamphetamine experienced similar severe sleep problems as women using heroin or other opioids. The study revealed gender differences in sleep problems only among methamphetamine users, with a higher proportion of poor sleeper women (He et al., 2020a, He et al., 2020b). Other study findings corroborated a higher prevalence of sleep disturbances in drug users, especially in heroin or other opioid users, compared to non-drug using healthy people. Additionally, users of heroin or other opioids experienced more severe sleep problems than methamphetamine users (Tang et al., 2015a, Tang et al., 2015b, Tang et al., 2015c).

Gender variations in sleep issues among users of industrial and traditional drugs remain inadequately explored, with existing findings presenting certain limitations for future research to address. The study also emphasizes the importance of in-depth analysis of inter-country and cross-cultural differences. Sleep problems are intricately linked to economic, social, and cultural patterns, with individuals of lower socioeconomic status facing a higher risk of sleep disturbances (Arber et al., 2009). This highlights the necessity for similar cross-cultural research in low and middle-income societies. The investigation delves into the quality and spectrum of sleep issues in the low-income country of Iran, encompassing both individuals with substance use disorders and healthy controls. Unlike studies that target specific populations, genders, or age groups, this approach is more comprehensive and generalizable.

## 3 Material and methods

The current research is a cross-sectional analytical study. The study population comprised all male and female individuals affiliated with Narcotics Anonymous (NA) groups in Bojnord city, Iran, in 2023. A total of 115 drug users (traditional: opioids/industrial: benzodiazepines, crack/cocaine, club drugs) were recruited through available sampling methods. Additionally, data from 140 healthy individuals were collected by referencing the same families to match demographic factors. Prior to data collection, written informed consent was obtained. While acquiring verbal informed consent from all participants, they were informed of their right to withdraw from the study at any time without penalty or adverse consequences, and confidentiality and anonymity were discussed with them. Inclusion criteria included drug use within the last 6 months, age between 20 and 55 years, and the absence of serious mental or medical illness. Exclusion criteria included reluctance to participate in the study. Eligible participants were invited to participate in the study after completing a 10-day detoxification treatment course. Then, demographic information and necessary details related to drug use were collected for all addicted men and women. Subsequently, the Pittsburgh Sleep Quality Index (PSQI) questionnaire was administered to evaluate sleep quality and sleep problems. Descriptive statistics such as mean, standard deviation, and variance were employed to analyze descriptive data. Furthermore, two-way analysis of variance and independent two-sample t-tests were utilized to test the hypotheses.

### 3.1 Measures

#### 3.1.1 Pittsburgh Sleep Quality Index (PSQI)

The questionnaire employed in this study comprises 9 questions and 7 scales, which are mental quality of sleep, delay in falling asleep, length of useful sleep, sleep adequacy (ratio of length of useful sleep to time spent in bed), sleep disorders (waking up at night), it measures the amount of sleep-inducing drug consumption and disruption in daily functioning (problems caused by insomnia during the day). The score of each scale is between 0 and 3, and a score of 3 in each scale indicates the maximum negative. The overall score of this questionnaire is considered between 0 and 21, and a score higher than 5 indicates inappropriate sleep quality (Mansouri et al., 2012). The reliability and validity of this questionnaire has been confirmed in various studies (Papi and Cheraghi, 2021; Torkian et al., 2021). In the present study, the test's reliability was established with a Cronbach's alpha of 0.78.

## 4 Results

The total sample size of the present study is 408 individuals. The sample comprises 140 healthy individuals (34.31%; male = 70, female = 70), 135 traditional drug users (32.49%; male = 69, female = 65), and 133 industrial drug users (32.49%; male = 66, female = 67). Additional demographic characteristics of the studied sample are reported in the table below.

Levene's test was employed to assess the homogeneity of variances (see Table 1). The results of the analyses pertaining to this test, as presented in Table 2, indicated that the variables of gender and group type (healthy individuals, industrial drug users, and traditional drug users) violated the assumption of homogeneity of variances. However, given the relatively small discrepancy in the variances between the two variables, this violation was deemed negligible, and the analysis proceeded with the assumption of homogeneity.

**Table 1:** Levels of drug use and their frequency among the study sample.

Characteristic		N = 60 (women)	N = 55 (men)
Any drug use: prior week	Benzodiazepines	10 (%16/66)	10 (%18/18)
	Crack/cocaine	10 (%16/66)	10 (%18/18)
	Opium	13 (%21/66)	10 (%18/18)
	Heroin	9 (%15)	9 (%16/36)
	Methamphetamine/Crystal	6 (%10)	7 (%12/72)
	Marijuana	9 (%15)	6 (%10/90)
	Club drugs (e.g., Ecstasy)	2 (%3/33)	2 (%3/63)
	Others	1 (%1/66)	1 (%1/81)
Any drug use: lifetime	Benzodiazepines	17 (%28/33)	17 (%30/90)
	Crack/cocaine	20 (%33/33)	18 (%32/72)
	Opium	23 (%53/33)	20 (%36/36)
	Heroin	19 (%31/66)	20 (%36/36)
	Methamphetamine/Crystal	10 (%16/66)	10 (%18/18)
	Marijuana	20 (%33/33)	18 (%32/72)
	Club drugs (e.g., Ecstasy)	13 (%21/66)	13 (%23/63)
	Others	7 (%11/66)	8 (%14/54)
Overdose experience	Overdose experience, prior 12 months^a^	11 (%18/33)	15 (%27/27)

a Refers to having personally overdosed.

**Table (2):** Levene's test for homogeneity of variances.

variables	N	Mean	Variance	F	df1	df2	Sig.
Contributor	408	1.9828	0.670	5.606	5	402	0.001
Full sample	408	1.4975	0.251				

P < 0.001.

The results presented in Table 3 demonstrate that the significance level of the test was 0.000, which is lower than the confidence level of 0.001. Therefore, it can be concluded that the variable of participant groups (healthy individuals, industrial drug users, and traditional drug users) in interaction with the gender variable has a positive and significant effect on the sleep quality of the participants. Additionally, the results of the analysis revealed that the significance level for investigating the difference in sleep quality in terms of the gender variable and the type of participant groups (healthy individuals, industrial drug users, and traditional drug users) was obtained as 0.02 and 0.000, respectively, which are less than 0.05 and 0.01. Consequently, it can be stated that the gender variable has a positive and significant effect on the sleep quality of the participants, and the sleep quality of individuals differs significantly across the participant groups (healthy individuals, industrial drug users, and traditional drug users). Furthermore, this analysis demonstrated that the variables of participant group type (healthy individuals, industrial drug users, and traditional drug users) and gender accounted for 29.8% and 2.3% of the variance in sleep quality, respectively.

**Table 3:** Two-way analysis of variance for sleep quality.

Dependent Variable: Overall quality of sleep						
Source	Type III Sum Of Squares	Df	Mean Square	F	Sig.	Partial Eta Squared
Corrected Model	1662.191^a^	5	332.438	39.025	. 001	0.327
Intercept	21463.664	1	21463.664	2.520E3	. 001	0.862
Contributor	1451.199	2	725.600	85.177	. 001	0.298
Full sample	80.175	1	80.175	9.412	0.002	0.023
Contributor * Full sample	139.260	2	69.630	8.174	. 001	0.039
Error	3424.515	402	8.519			
Total	26272.000	408				
Corrected Total	5086.706	407				
A. R Squared = 0.327 (Adjusted R Squared = 0.318)						

P < 0.001.

As evident from Table 4, which presents the results of multiple comparisons analysis (Tukey's test), industrial drug users exhibit the largest mean difference of 3.9554 with the healthy individuals group, with a significance level of 0.000. This finding indicates that the most substantial difference in sleep quality, with respect to the gender variable, can be observed in the industrial drug user group. Additionally, according to the graph (1), it can be stated that in terms of group differences, the most pronounced effects on sleep quality for both men and women can be seen among the industrial drug user group, followed by the traditional drug user group.

**Table 4:** Multiple comparisons (Tukey's test) for sleep quality across groups.

Overall quality of sleep Tukey HSD						
(I) contributor	(J) contributor	Mean Difference (I-J)	Std. Error	Sig.	95% Confidence Interval	
					Lower Bound	Upper Bound
health	Industry	−3.5495^a^	0.35206	. 001	−4.3777	−2.7213
	traditional	−4.2816^a^	0.35341	. 001	−5.1129	−3.4502
industry	Health	3.5495^a^	0.35206	. 001	2.7213	4.3777
	traditional	−0.7321	0.35658	0.101	−1.5709	0.1067
traditional	health	4.2816^a^	0.35341	. 001	3.4502	5.1129
	industry	0.7321	0.35658	0.101	−0.1067	1.5709

Based on observed means.

The error term is Mean Square(Error) = 8.519.

a The mean difference is significant at the .05 level.

Table 5 demonstrates that, in general, women exhibited poorer sleep quality than men across all three groups, although this difference reached statistical significance in only one group. More specifically, the average score of the total sleep quality between women and men in the healthy group (control) and the group of traditional drug users is not significant, but a significant difference was found between the sleep quality of women and men in the group of industrial drug users. This means that the quality of women's sleep in the group of industrial drug users is weaker than that of men in this group. It was also significantly observed that women and men in the groups of traditional and industrial drug users had a much higher mean in seven scales and overall score of sleep quality (weaker sleep quality) than healthy people.

**Table 5:** Gender differences in Sleep Quality and its seven subscales across groups.

Variables	Control group (healthy)			Drug users group					
				Industrial drug users			Traditional drug users		
	Man	Woman	Sig	Man	Woman	Sig	Man	Woman	Sig
Mental quality of sleep	0,6000 (±0,54904)	0,8143 (±0,62073)	0,032	1, 2059 (±0,70306)	1,7015 (±0,77879)	0,000	1,4118 (±0,77720)	1,7015 (±0,77879)	0,032
Delay in falling asleep	0,7286 (±0,56264)	0,9143 (±0,60775)	0,063	1, 2647 (±0,72519)	1,7612 (±0,76057)	0,000	1,5441 (±0,90494)	1,6418 (±0,81094)	0,510
Sleep duration	0,9429 (±0,61115)	0,6268 (±0,54298)	0,002	0, 8088 (±0,62908)	1,3284 (±0,72589)	0,000	1,0588 (±0,89580 (	0,8806 (±0,6160)	0,180
Enough sleep	0,4286 (±0,49844)	0,4286 (±0,62720)	1000	0,7353 (±0,56298)	1,2239 (±0,69240)	0,000	0,8088 (±0.0,67487)	0,8060 (±0,67955)	0,981
Sleep disorder	0,7571 (±0,600038)	0,7286 (±0,56264)	0,772	0, 9118 (±0,61657)	1,3582 (±0,71141)	0,000	1,3088 (±0,93453)	0,8060 (±0,63338)	0,000
Need for sleeping pills	0,2857 (±0,68404)	0,1143 (±0,32046)	0,060	0,6029 (±0,55016)	0,5821 (±0,55457)	0,827	1,3676 (±0,84473)	1,3134 (±0,58281)	0,665
Morning sickness	0,9286 (±0,59761)	1,0429 (±0,66889)	0,288	1,3088 (±0,73824)	1,5970 (±0,79886)	0,031	1,4265 (±0,60634)	1,5075 (±0,56066)	0,422
Overall quality of sleep	4,6000 (±2,68502)	4,6714 (±2,69944)	0,876	6,8382 (±3,22725)	9,5522 (±3,26725)	0,000	8,9265 (±2,45750)	8,9552 (±2,2592)	0,944
**-**	P > 0.001			P < 0.001			P > 0.001		

Upon examination of the statistical analysis results presented in Table 6, and considering that the significance level of the test for the sleep quality variable and its components (with the exception of ‘The need for sleeping pills') was obtained above the confidence level of 0.001, it can be concluded that industrial and traditional drug users do not exhibit statistically significant differences from each other in terms of overall sleep quality and its various components (excluding ‘The need for sleeping pills'). This finding suggests that the type of drug used (industrial or traditional) may not be a determining factor in the overall sleep quality experiences of drug users.

**Table 6:** Independent two-sample *t*-test to comparison of Sleep quality components between industrial and traditional drug users.

Variable	Levine's test to check the homogeneity of variances		t	df	sig (ttest)	Mean Difference	95% confidence interval of the difference	
	f	sig					Lower	Upper
Mental quality of sleep	0.011	0.916	−1.089	265.745	0.277	−0.10454	−0.29359	0.08452
Delay in falling asleep	1.306	0.254	−0.900	262.690	0.369	−0.09039	−0.28821	0.10743
Sleep duration	0.626	0.429	1.226	264.631	0.221	0.11178	−0.06781	0.29137
Enough sleep	2.982	0.085	2.095	265.867	0.037	0.17327	0.01045	0.33608
Sleep disorder	0.326	0.568	−0.690	262.557	0.491	−0.06216	−0.23960	0.11529
Need for sleeping pills	4.308	0.039	−9.457	245.912	0.000	−0.74575	−0.90107	−0.59043
Morning sickness	12.673	0.000	−0.170	266	0.865	−0.01431	−0.18022	0.15159
Overall quality of sleep	22.830	0.000	−1.901	266	0.058	−0.73211	−1.49028	0.02606

P > 0.001.

Based on the results presented in Table 7 and considering that the significance level of the test was 0.001, which is less than the confidence level of 0.01, it can be concluded that drug users (both traditional and industrial) show statistically significant differences with healthy people in terms of the overall quality of sleep and its various components. This means that healthy people show a more favorable sleep quality profile compared to drug users. These findings emphasize the fundamental impact of drug use on sleep patterns and quality, regardless of whether the drugs used are classified as traditional or industrial.

**Table 7:** Independent two-sample *t*-test to comparison of Sleep quality components between drug users and healthy individuals.

Variable	Levine's test to check the homogeneity of variances		t	df	sig (*t*-test)	Mean Difference	95% confidence interval of the difference	
	f	sig					Lower	Upper
Mental quality of sleep	20.163	. 001	−10.522	406	. 001	−0.79659	−0.94541	−0.64776
Delay in falling asleep	35.819	. 001	−9.385	406	. 001	−0.73454	0.07826	−0.88840
Sleep duration	0.058	0.809	−3.314	340.603	0.001	−0.22548	0.06804	−0.35931
Enough sleep	0.001	0.974	−7.318	331.136	001	−0.46322	0.06330	−0.58774
Sleep disorder	1.603	0.206	−6.334	344.529	001	−0.42132	0.06652	−0.55216
Need for sleeping pills	7.967	0.005	−11.855	364.921	001	−0.76269	0.06434	−0.88920
Morning sickness	24.741	0.000	−6.768	406	001	−0.47324	0.06992	−0.61069
Overall quality of sleep	2.514	0.114	−13.128	325.315	001	−3.91279	0.29806	−4.49916

P < 0.001.

## 5 Discussion

According to our comprehensive literature review, this study represents the first investigation in Iran to evaluate gender differences in sleep problems among traditional and industrial drug users. The overall prevalence of sleep problems (PSQI>5) between men and women using traditional drugs was 75.1 and 75.6%, in industrial drug users 69.0 and 77.0%, and in healthy men and women 28.8 and 26.1%. The findings also underscore a consistent trend across all three groups, revealing that female participants consistently exhibited inferior sleep quality compared to their male counterparts. However, statistical significance for this difference was observed in only one group. This aligns with prior research emphasizing the intricate interplay between gender dynamics and sleep quality (Glavin et al., 2021; Li et al., 2021; Madrid-Valero et al., 2017). The study contributes valuable insights into the nuanced relationship between drug use, gender disparities, and sleep quality, thus fostering a deeper understanding of mental health implications within this context.

It is well-established that the prevalence of sleep problems in the general population is consistently higher among women compared to men (39–41). In the meta-analysis study by Alimoradi et al. (2022) the prevalence of sleep problems between males and females worldwide are provided. The estimate of sleep problems was calculated based on the reports emanating from 15 countries for the female subgroup and 13 countries for the male subgroup with nearly 115,000 participants. The results showed that the prevalence of sleep problems was 24% for female participants and 27% for male participants (Alimoradi et al., 2022). The findings of a population-based study show that women have poorer sleep quality than men, but little gender difference was observed in the prevalence of insomnia (Tang et al., 2017a, Tang et al., 2017b). Also, the findings of a population-based regional survey in Saudi Arabia report the prevalence of obstructive sleep apnea in men and women, respectively, 1.4 and 1.8 (Wali et al., 2017).

Gender emerges as a crucial determinant in shaping sleep behavior, influencing sleep architecture, and modulating the prevalence and manifestation of sleep disorders (Haufe and Leeners, 2023). Several factors are involved in reducing sleep quality and increasing sleep disorders among women. One of these factors may be attributed, in part, to their proclivity for engaging in dysfunctional cognitive processes, specifically heightened levels of rumination and worry, relative to men (Nolen-Hoeksema and Aldao, 2011; Jose and Brown, 2007). Rumination and worry play an essential role in a wide range of people in society (Erickson et al., 2020), including people with sleep disorders (Olatunji et al., 2023), people with other mental disorders (Takano et al., 2012) and healthy people (Tang et al., 2023). For example, Harvey's cognitive model of insomnia postulates that excessive worry about sleep and the consequences of poor quality sleep causes spontaneous arousal, emotional distress, and excessive monitoring of thoughts that threaten sleep quality in women (eg, insufficient sleep indicators or poor daytime functioning), which ultimately lead to actual sleep loss. Thus, gender differences in worry cause some factors that are important in the development and maintenance of sleep disorder (Harvey, 2002).

Another significant factor contributing to the explanation of gender disparities in pretreatment symptoms is the fluctuation of hormones associated with female physiology. The menstrual cycle is the main source of hormonal fluctuations of estradiol and progesterone over a 4-week period. The level of estradiol and progesterone is initially low (follicular phase), with a cycle peak in estradiol indicating ovulation, followed by a rise in estradiol and progesterone in the second half of the cycle before declining to an initial low (luteal phase). In support of this notion, adult women report poorer sleep quality during hormonal periods, including the luteal phase of the menstrual cycle, the perinatal period, and menopause (Suh et al., 2018; Mong and Cusmano, 2016).

In addition to the aforementioned factors, a range of other psychosocial stressors impacting sleep are mood disorders, heightened susceptibility to adverse socioeconomic factors, and gender-based discrimination, particularly among women from racial-ethnic minority backgrounds (Benges et al., 2024.,). Also, gender expectations may contribute to a reduction in sleep duration. Venn et al., (2008) assert that women are both expected and expect themselves to manage the emotional and practical needs of family members during the night. Their study, examining 26 British couples of working age with children, revealed that women experienced more frequent awakenings to attend to emotional and caregiving responsibilities. Another study of 25 dual-income working-class couples under 50 in the United States found that women reported more sleep interruptions related to caregiving, even when working night shifts (Maume David et al., 2009). Additionally, a nationally representative study of American working-age parents indicated that women experienced significantly more sleep interruptions for caregiving purposes, independent of work and parenting responsibilities (Burgard, 2011). The correlation between sex-related hormonal fluctuations and the occurrence of insomnia and sleep disorders, potentially influenced by these hormonal changes, requires further exploration.

Furthermore, the present study revealed that a disparity in sleep quality between genders is observed exclusively among industrial drug users. Surprisingly, this distinction was not observed among healthy individuals and traditional drug addicts, contrary to initial expectations. That is, out of the three studied groups, only women who used industrial drugs had poorer sleep quality than men. This finding is in line with some of the results of previous studies ((He et al., 2020a, He et al., 2020b; Tang et al., 2017; Wali et al., 2017).

Specifically, concerning the findings of the current study—namely, the decreased sleep quality observed in addicted women—it can be concluded that gender disparities exist in the impact of acute industrial drug use on reward-related mental and behavioral qualities (Perez et al., 2008) may contribute to the gender difference in sleep problems, that is, women who use industrial drugs compared to their peers (in the healthy group and the group of traditional drug users) show poorer sleep quality and higher prevalence of sleep problems. Gender interactions with sleep quality can also be more complex. Even a single intranasal dose of industrial drugs can cause a significant reduction in subjective and objective sleep quality measurements (Lin et al., 2003). Also, gender differences in sleep quality between men and women who use synthetic drugs this may be due to the gender-specific role of certain genes in industrial drug use disorder (Hellem et al., 2015) or the fact that this group of women experiences higher rates of depression than their counterparts in other groups (AlRyalat et al., 2021). Furthermore, compared with male MA users, although female users seem more dependent to MA, they show diminished dopamine responses and fewer cases of emergency department-related deaths involving MA (Dluzen and Liu, 2008). Previous research showed that, Poor sleep quality is associated with increased use of cocaine in women (Dolsen and Harvey, 2017) and methamphetamine relapse (McGregor et al., 2005). Also the results of a recent study on female monkeys showed that Methamphetamine dose-dependently disrupted actigraphy-based sleep parameters. Treatment with either suvorexant or MK-1064 dose-dependently improved actigraphy-based sleep in monkeys treated with methamphetamine. Additionally, both suvorexant and MK-1064 promoted actigraphy-based sleep in a group of monkeys with baseline short actigraphy-based sleep (Borgatti et al., 2024). Additional results of the present study revealed that in general, healthy people had better sleep quality compared to drug users (in both traditional and industrial groups). Previous studies have shown a much higher prevalence of sleep problems in drug users than in healthy individuals (He et al., 2020; Tang et al., 2015a, Tang et al., 2015b, Tang et al., 2015c).

## 6 Conclusion

This study represents the first investigation among drug-using men and women in Iran. In the meantime, during comparisons with other studies conducted outside Iran, it was observed that the level and amount of relationships obtained in this study are somewhat different from previous studies. At the same time, the comparison results of each of the subscales of sleep quality and the total score of this scale are consistent with the results of some studies. The disparities identified in this study prompt further inquiry into the unique socio-cultural and environmental factors that may modulate the relationship between drug consumption and sleep quality in the Iranian context. Additionally, this research lays the foundation for future investigations, encouraging a more nuanced and context-specific understanding of sleep quality dynamics among individuals with and without a history of drug use, ultimately fostering targeted interventions to enhance both physical and mental well-being within this demographic.

## 7 Limitations

This study faced several constraints, primarily rooted in the context of North Khorasan province, Iran. Firstly, the disproportionately higher prevalence of drug use and mortality rates among men, as compared to women, resulted in a smaller percentage of female participants in the sample. Additionally, the lower percentage of smoking and alcohol consumption among women, even though associated with sleep-related problems, may suggest other contributing factors not considered in this study. Future research is encouraged to explore these factors. The study did not evaluate various aspects related to sleep problems, such as socioeconomic status, drug use patterns, severity of dependence, and individual histories. Examining sleep problems in early childhood as predictors of later alcohol and drug-related issues is also recommended. The study focused solely on drug addiction and did not consider other behavioral addictions, such as Internet addiction, which might also be linked to sleep problems. Future investigations should explore gender differences in sleep related to Internet addiction and virtual networks. The study did not include individuals of the third gender, and it is recommended that future research addresses this gap in understanding the sleep patterns of this minority group. Lastly, it is important to note that all participants were in a recovery problems and were distinct from the general drug users.

## CRediT authorship contribution statement

**Mohammad Khorrami:** Writing – review & editing, Writing – original draft, Software, Methodology, Formal analysis, Conceptualization. **Fatemeh Khorrami:** Software, Investigation, Conceptualization. **Kosar Haghani:** Methodology, Investigation. **Farshid Fathy Karkaragh:** Software, Funding acquisition, Data curation. **Ayda Khodashenas:** Writing – original draft, Resources. **Sara Souri:** Resources, Investigation, Formal analysis.

## Declaration of competing interest

The authors declare that there is no conflict of interest in this research.

## Data Availability

Data will be made available on request.
